# Immediate Loading Implant-Supported Fixed Full-Arch Rehabilitation Using a New Clinical Decision-Support System: A Case Series

**DOI:** 10.7759/cureus.67879

**Published:** 2024-08-26

**Authors:** Ahmed Bahaa, AbdAllah Bahaa, Nada El-Bagoury, Nora Khaled, Wael A El-Mohandes, Ahmed M Ibrahim

**Affiliations:** 1 Oral and Maxillofacial Surgery, Royal College of Surgeons of Edinburgh, Edinburgh, GBR; 2 Oral and Maxillofacial Surgery, Faculty of Dental Medicine, Al-Azhar University, Cairo, EGY; 3 Orthodontics, Faculty of Dental Medicine, Al-Azhar University, Cairo, EGY; 4 Orthodontics, Faculty of Dentistry, Misr International University, Cairo, EGY; 5 Orthodontics, Faculty of Dentistry, Ain Shams University, Cairo, EGY; 6 Research and Development, Innovinity Medical Hub, Cairo, EGY; 7 Endodontics, Faculty of Dentistry, Cairo University, Cairo, EGY

**Keywords:** peri-implant marginal tissue health, carames classification, full-arch rehabilitation, immediate functional loading, immediate implant

## Abstract

Background

Implant-supported full-arch rehabilitation is an effective treatment for edentulous patients. It restores mastication, facial aesthetics, and psychological well-being. Patient-related outcome measures support the validity of this approach, emphasizing the importance of effective prosthodontic interventions for this patient population. This study aims to present a case series for fixed implant-supported full-arch rehabilitation using the new Carames classification (CC).

Methods

A total of seven patients with generalized periodontitis or non-restorable multiple teeth were indicated for extraction and replacement with a fixed full-arch implant-supported prosthesis. According to the Carames classification, most cases were categorized as CCI or CCII classes for both the upper and lower jaws. Before the surgery, screw-retained provisional complete dentures were constructed and adjusted for the vertical occlusal dimension and smile lines. After the extractions, 70 implants were immediately placed in one or both arches for the seven patients, followed by bone grafts with the dual-zone grafting technique. Multi-unit abutments were then placed and welded to a metal bar for stable fixation. The provisional denture was fitted snugly over the metal bar for immediate functional loading. After three months of healing, it was used as a biocopy to fabricate the final prosthesis. The implant loss and the peri-implant marginal tissue health status were assessed annually for three years. Statistical analysis compared the marginal bone loss as a change from the baseline over the year.

Results

No implant or prosthesis loss was reported over the three years. Peri-implant marginal tissue health showed promising results without bleeding and suppuration on probing and probing depths between 3 and 3.5 millimeters. Marginal bone loss was minimal over the three years, with some cases showing bone gain.

Conclusion

Using the Carames classification as a clinical decision support system in implant-supported full-arch rehabilitation showed promising results in peri-implant tissue health and no implant loss during three years of follow-up. The implant placement and prosthesis fabrication protocol in this study could be valuable for further research.

## Introduction

Edentulism, which refers to losing most or all teeth, is a significant global oral health issue. It is the ultimate indication of the disease burden for oral health and affects millions of individuals worldwide. The occurrence of edentulism differs widely, with up to 70% of people aged 60 or older being affected [1]. Some epidemiological studies indicate a decrease in occurrence in developed countries due to preventive oral health measures. However, the increase in life expectancy appears to offset this trend and promote the need for treatment [2,3]. Whether total or partial, prolonged edentulism can lead to the progressive resorption of alveolar processes and cause many dental problems.

Implant-supported fixed full-arch rehabilitation (IFFR) represents an effective treatment modality for edentulous patients. This intervention offers a reliable means of restoring masticatory function, facial aesthetics, and psychological well-being. The validity of this prosthodontic approach is supported by patient-related outcome measures, which are evaluated through oral health-related quality of life parameters, patient satisfaction, and preference. The significance of these variables underscores the need for effective prosthodontic interventions in this patient population [2,4].

Implant-supported fixed prosthesis has a high success rate with different placement and loading protocols. Immediate or early functional loading with immediate placement of dental implants can be just as effective as traditional loading protocols. The immediate or early loading protocols demonstrate comparable implant survival and marginal bone loss in fixed implant-supported prostheses for edentulous patients receiving treatment for maxillary and mandibular arches with at least three years of follow-up or more [5,6].

The use of clinical decision support systems (CDSS) can be highly beneficial for medical professionals in the fields of diagnostics, treatment planning, and rehabilitation. These systems can assist in making informed treatment decisions based on patient-specific diagnostic findings [7]. In 2019, Carames developed a comprehensive CDSS for implant-supported fixed prostheses treatment options [8]. The system provides various treatment options for the edentulous maxilla and mandible, considering the anatomical level and pattern of atrophy of the alveolar process and patient-specific risk factors. It uses a decision-making process to select a specific treatment option from predefined implant rehabilitation and surgical workflow schemes. Patient clinical information is crucial in making implant-prosthodontic decisions. Proper consideration of medical history, expectations, biomechanics, and prosthodontic design is vital for optimal results, as proposed by the 2019 CDSS [8].

According to a retrospective study conducted in 2021 using the Carames CDSS, it was found that the level of alveolar atrophy did not affect the survival rates of implants in the mandible. However, it did impact the survival rates of implants in the maxilla, which decreased with an increase in the level of atrophy. Despite this, a high percentage of implant survival (over 97%) was observed after two to five years of follow-up [9]. To our knowledge, no studies were conducted to assess the recently introduced implant dentistry core outcome set and measurements (ID-COSM) [10] in the context of an implant-supported fixed prosthesis implementing the new Carames CDSS for fixed full arch rehabilitation. Therefore, the current study aimed to present a case series for rehabilitating partial or complete edentulous arches using the new Carames CDSS with immediate loading implant-supported fixed prosthesis and a three-year follow-up assessing the newly introduced ID-COSM.

## Materials and methods

Methods

The following case series involved seven patients with masticatory and aesthetic issues in one or both arches due to generalized periodontitis and multiple non-restorable teeth. These patients required extraction and full-arch rehabilitation with immediate implant placement and immediate functional loading. These patients received treatment at our private dental center, Innovinity Medical Hub, located in Heliopolis, Cairo, Egypt, from 2020 to 2023. The average age of the patients was 51.57 ± 7.5, with four males and three females. Three patients had controlled hypertension and diabetes, while the others did not have any systemic medical conditions. Four male patients were smokers, whereas females were not. A total of 70 implants were placed in the seven cases presented.

All patients underwent preoperative cone beam CT scans and had extraoral and intraoral photographs taken. They received the same surgical procedures, with modifications for different treatment options as required. Before surgery, impressions of the maxilla and mandible were made, and diagnostic casts were poured, establishing vertical occlusal dimension and smile lines to facilitate the construction of a provisional denture to act as a functional immediate loading prosthesis after implant placement. Betadine was applied to the surgical site for five minutes, and then all the patients received treatment under local anesthesia with articaine 4% and adrenaline (Septanest). Atraumatic extraction using periotomes and piezotome was performed, followed by curettage of the extraction sockets and application of Garamycin. A mucosal flap approach was used, and the implants were drilled into the chosen sites at equal distances. The used implant was a tapered Vitronex implant using a Morse taper connection and an internal hex. The implants were placed according to the manufacturer's instructions, achieving primary implant stability of 40 to 45 Ncm. After implant placement, Cerabone xenograft (Botiss) mixed with Garamycin was placed using the dual-zone grafting technique [11]. Following grafting, multi-unit abutments were placed and screwed to the metal sleeves to be welded with an intraoral welder (JDental Care) to a metal bar for fixation. The mucosal flap was sutured using loose, simple, interrupted sutures with a 4-0 VICRYL suture.

**Figure 1:**
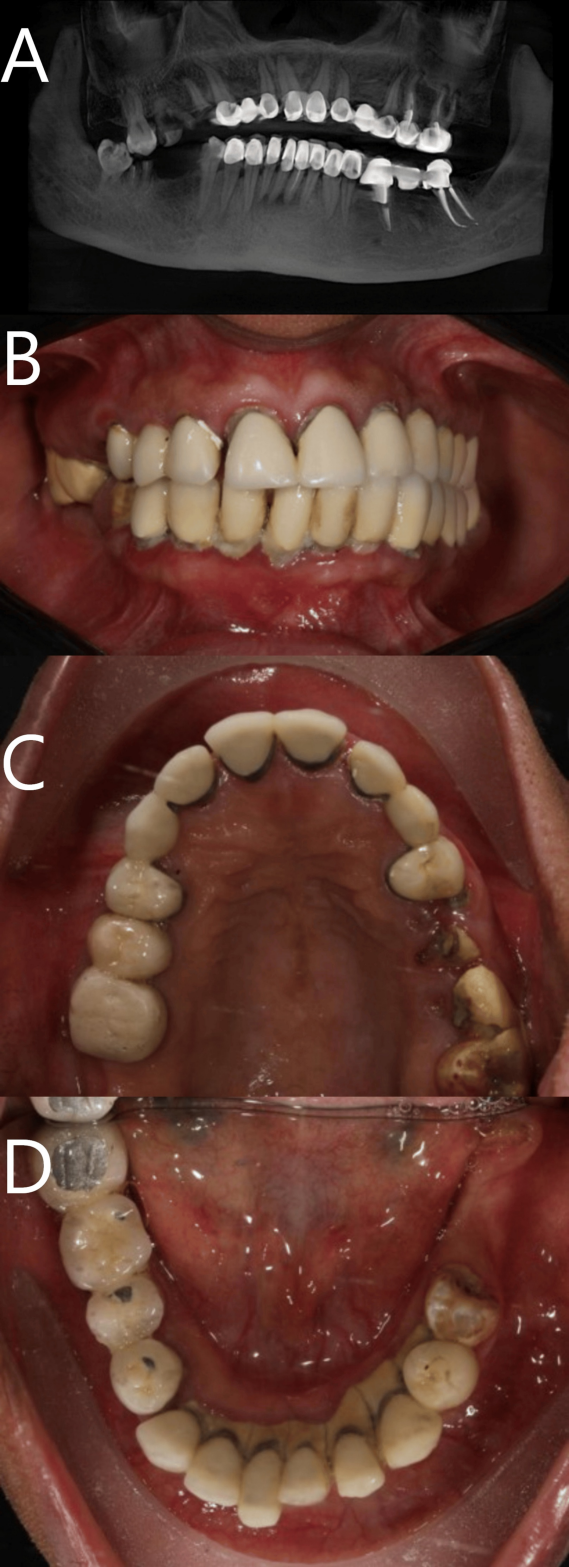
Preoperative CBCT scan (A) and intraoral photographs of the upper and lower jaws (B-D) show a failed long-span bridge with generalized periodontitis and non-restorable teeth.

**Figure 2:**
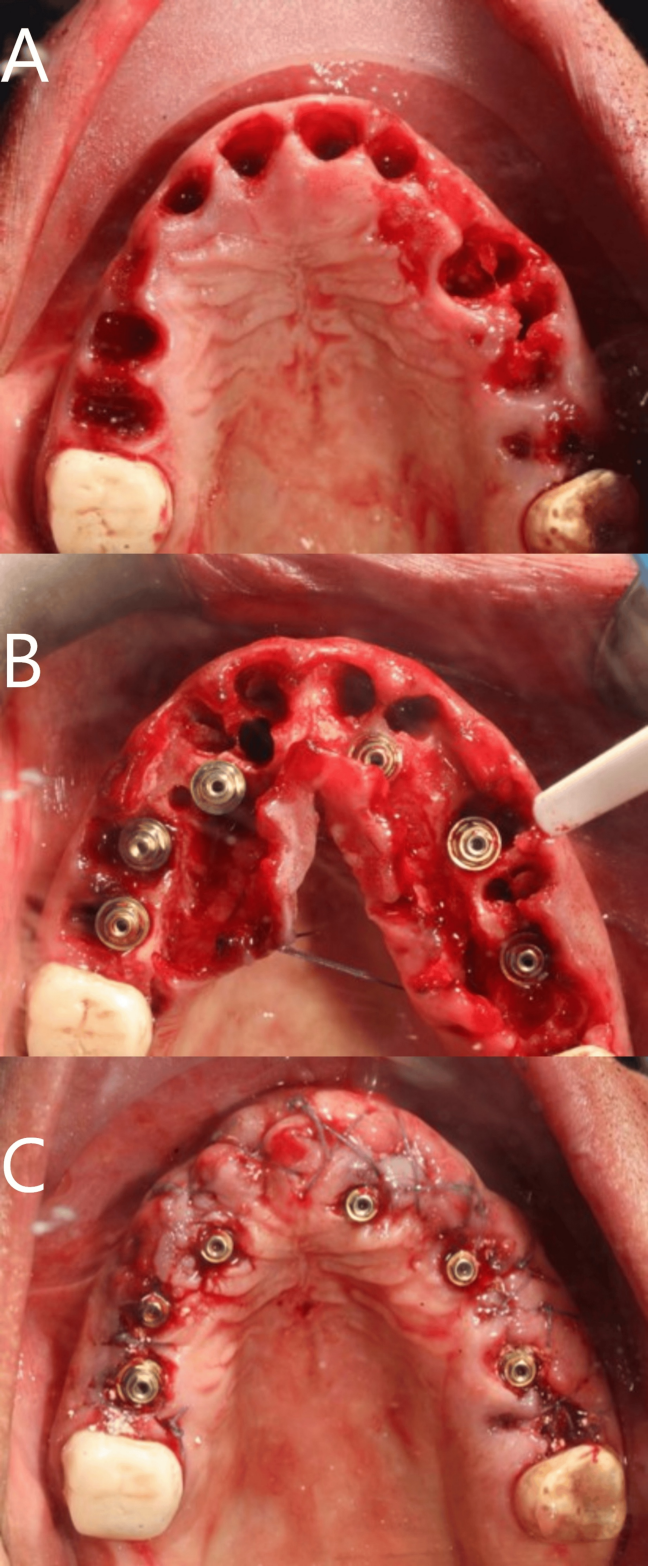
Surgical steps in the upper jaw. (A) after extraction, (B) after implant placement, and (C) suturing.

**Figure 3:**
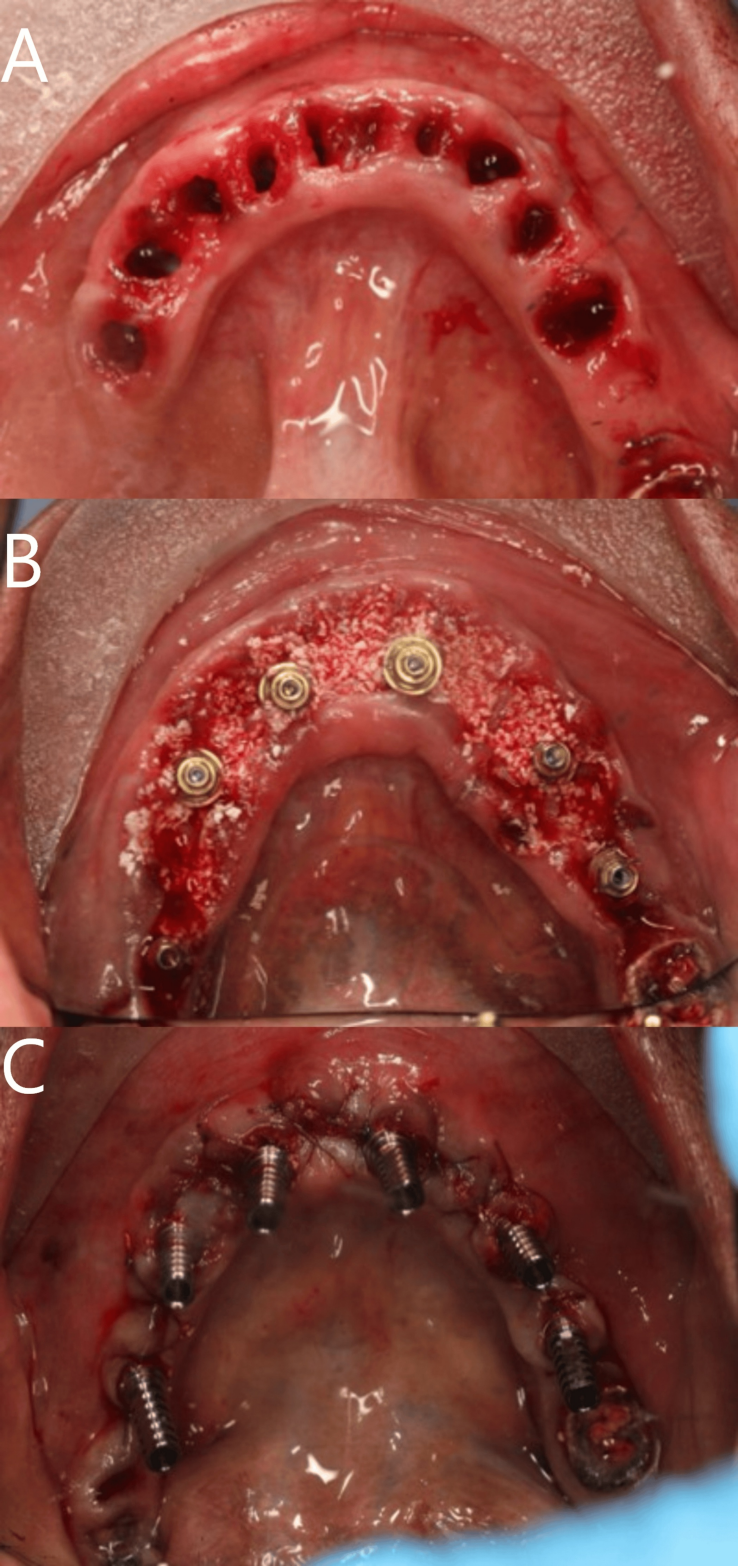
Surgical steps in the lower jaw. (A) after extraction, (B) after implant placement and bone graft, and (C) suturing.

**Figure 4:**
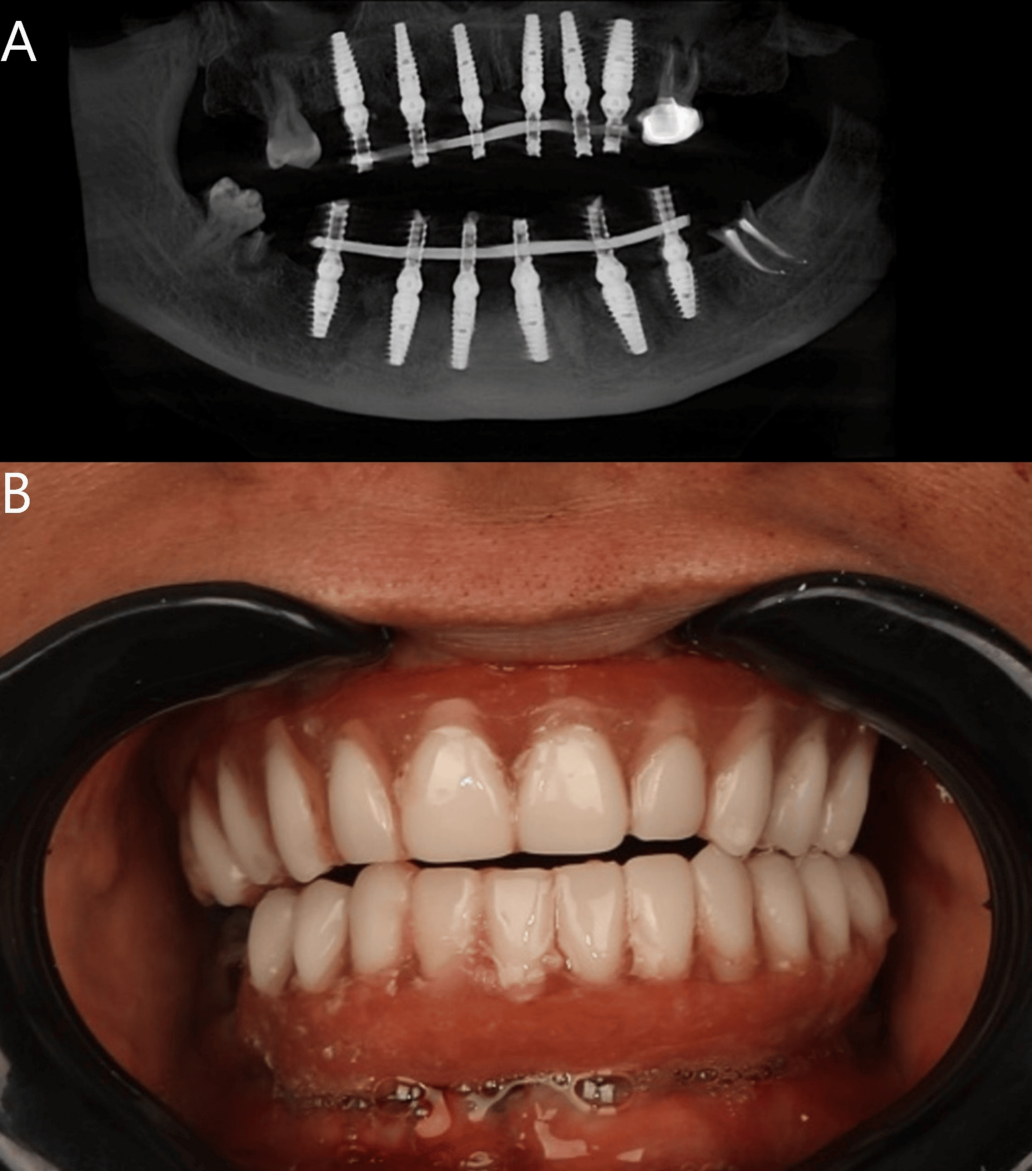
CBCT scan showing welded multi-unit abutment over implants (A) and intraoral photograph of the provisional denture in place as immediate functional loading (B).

**Figure 5:**
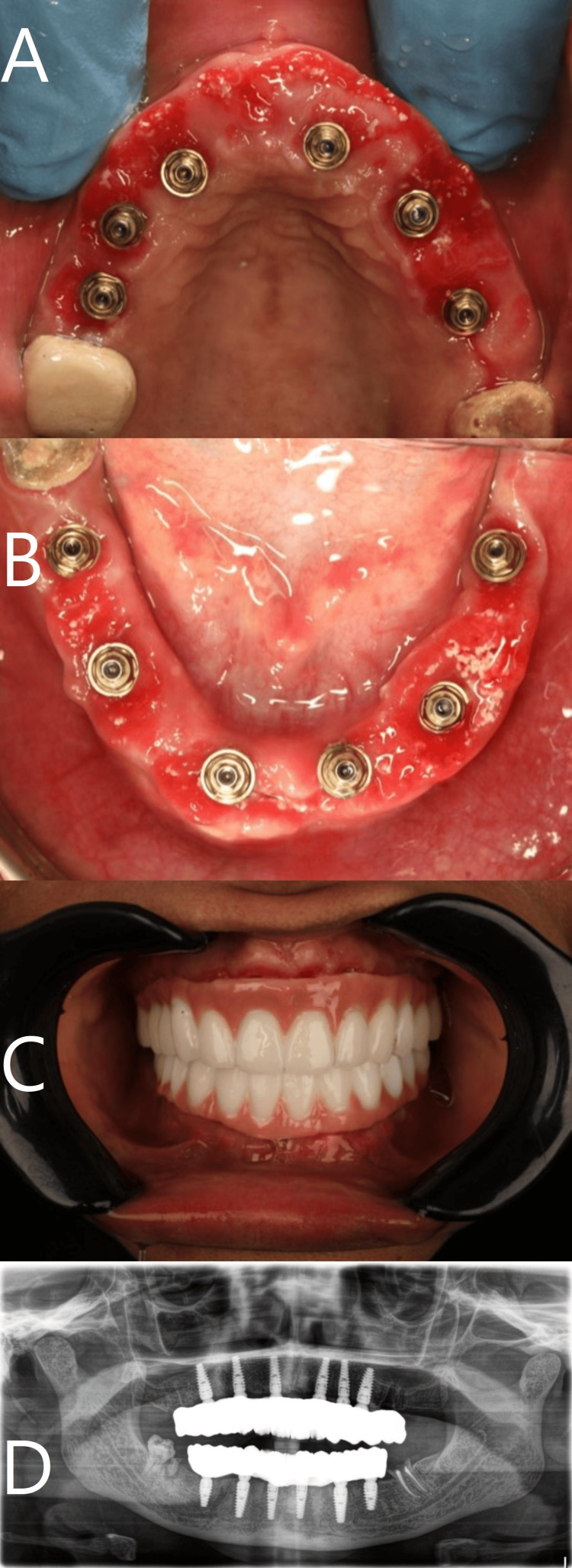
Intraoral photograph for healing after three months for upper and lower jaws respectively (A, B), final prosthesis in place (C), and postoperative panoramic X-ray (D).

The provisional screw-retained denture was fabricated to fit a metal bar and sleeves, snugly compress the gingival tissues with no gaps, and placed immediately for functional use. The patient was prescribed a broad-spectrum antibiotic and NSAIDs and advised to use chlorhexidine mouthwash twice daily for oral hygiene. Sutures were removed after one week, and the patient was scheduled for a follow-up visit in three months to monitor healing progress. A digital scan of the soft tissue and implant abutment was taken using scan-bodies. The provisional denture was scanned as a biocopy to create the final prosthesis. A polymethyl methacrylate framework was used for a try-in before constructing the final prosthesis. The final prosthesis was made from cutback monolithic zirconia, with pink porcelain used to replicate gingival tissues in all cases except for one where a titanium framework was used with zirconia crowns. The final definitive prosthesis was a screw-retained FP3 zirconia prosthesis. A detailed case is shown in Figures 1-5.

Outcomes

All the patients were followed up yearly for three years after the definitive final prosthesis placement. The outcomes evaluated followed the newly introduced ID-COSM, focusing on post-operative complications and peri-implant marginal tissue health status. The outcomes were assessed at T0 (baseline after prosthesis delivery), T1 (after one year), T2 (after two years), and T3 (after three years). The health of peri-implant marginal tissue was evaluated using three criteria: the presence or absence of bleeding or suppuration on probing, probing pocket depth (PPD), and marginal bone level. PPD was measured circumferentially using a 0.5 mm periodontal probe at four sites per implant, and the mean of all sites was reported. Marginal bone level was measured from the implant platform to the bone level in mesial and distal sites on cone beam computed tomography scans after unscrewing the implant-supported prostheses. Marginal bone loss was calculated based on changes from baseline over time. All measurements were recorded in millimeters.

Statistical methods

The statistical significance level was set at 5%. Statistical analysis was performed using R and R Studio software [12]. The data was organized, manipulated, and summarized using the "tidyverse" R package. Continuous data was summarized using mean and standard deviation or median and range. Data was checked for normality using the Shapiro-Wilk test from the “rstatix” R package. Graphs were created using the "ggpubr" R package. The mean values of marginal bone loss were compared using the Friedman test, and a two-way mixed ANOVA was used to evaluate the interaction of smoking and time on marginal bone loss values. A significant Friedman test is followed by a pairwise Wilcoxson signed-rank test to identify significant comparisons. P-values are adjusted using the Bonferroni multiple-testing correction method.

## Results

**Figure 6:**
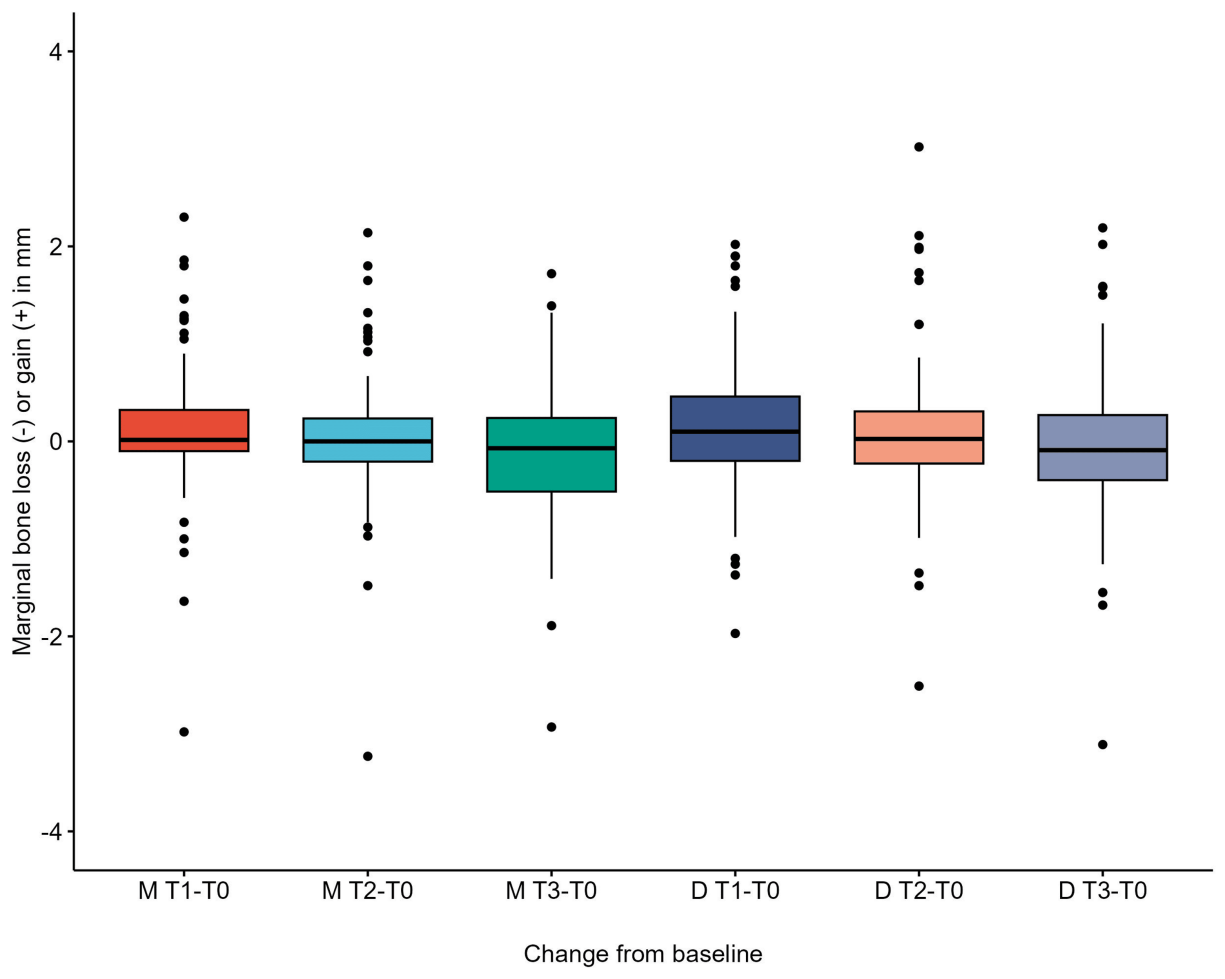
Boxplot showing marginal bone loss at different time points.

**Figure 7:**
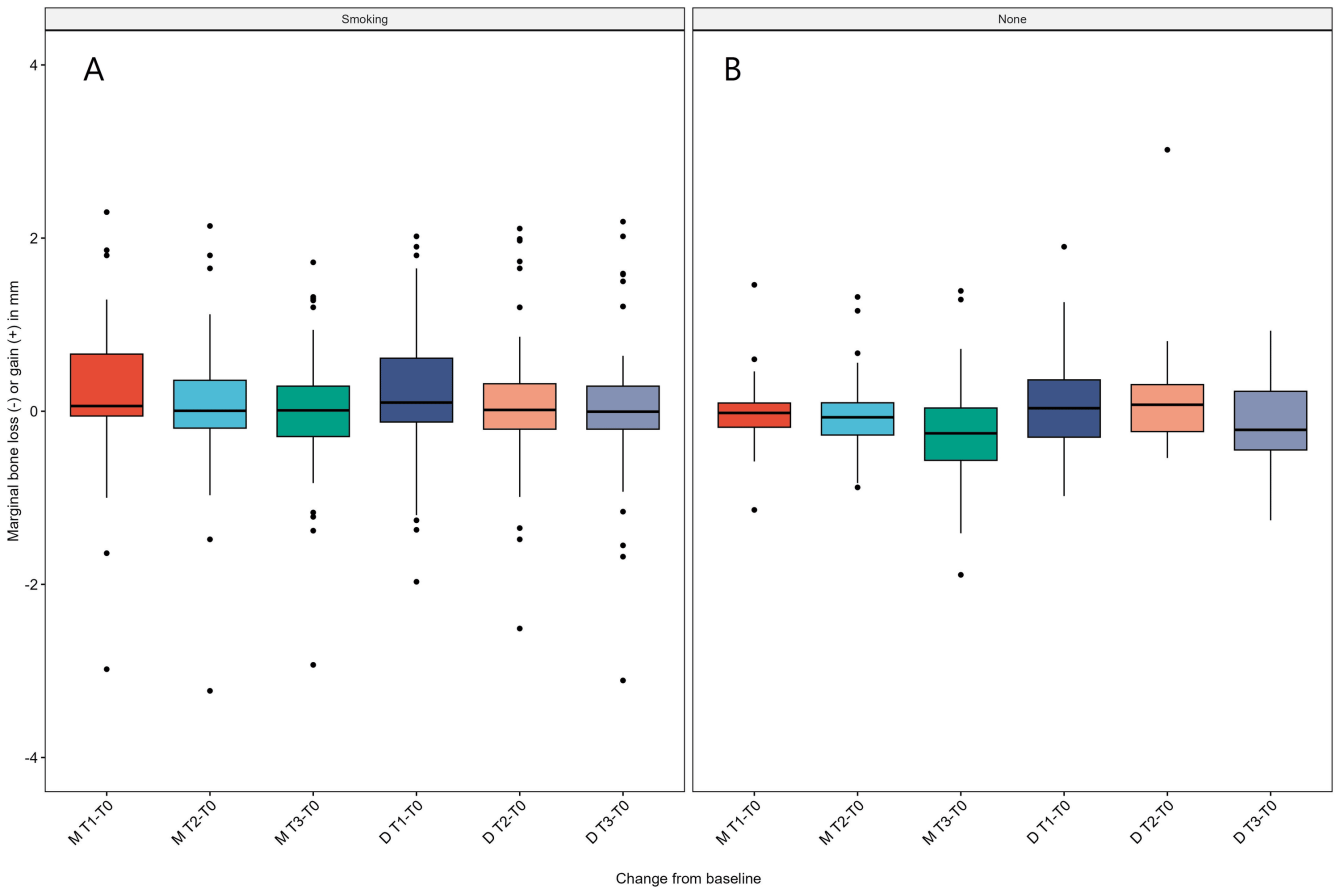
Boxplot showing marginal bone loss values concerning smoking patients (A) and none (B).

The patients' Carames CDSS classification, arch position, and implant numbers are shown in Table 1. After the 70 implants were placed in 7 full-arch cases and followed up yearly for three years, there were no reported surgical complications or implant or prothesis losses. Peri-implant marginal tissue health has shown promising results, with no bleeding or suppuration on probing and PPD ranging from 3 mm to 3.5 mm for all implants during various follow-up periods. Marginal bone loss values in mesial and distal sites (change from baseline) are shown in Table 2 and Figure 6. The effect of smoking on marginal bone loss is shown in Figure 7.

**Table 1:** Carames classification for arches and the number of implants used in each case. CC: Carames classification type.

Case #	Arch to receive IFFR	Carames classification	Treatment option	Number of implants	Notes
Case #1	Maxillary	CC1 (left side) CC2 (right side)	A: 6 to 8 straight equidistant implants	8	Closed sinus lift using Densah bur in the upper right-most posterior implant.
Case #2	Maxillary	CC2	B: four straight implants in the area limited by the anterior wall of the maxillary sinus and two tilted posterior implants	6	The upper right-most posterior implant tilted by 17 degrees.
	Mandibular	CC1	A: 6 to 8 straight equidistant implants	6	None
Case #3	Maxillary	CC1 (left side) CC2 (right side)	A (left) B (right)	6	The upper right-most posterior implant tilted by 17 degrees in option B.
Case #4	Maxillary	CC2	B: four straight anterior implants and two tilted posterior implants	6	The posterior implants are tilted at 15 and 17 degrees on the right and left sides.
	Mandibular			6	The posterior implants are tilted at 30 and 25 degrees on the right and left sides.
Case #5	Maxillary	CC1	A	8	None
Case #6	Maxillary	CC1	A	6	None
	Mandibular			6	
Case #7	Maxillary	CC5	B: four short implants in the maxillary anterior region and two zygomatic implants	6	Two zygomatic implants were placed, one on each side.
	Mandibular	CC2	A	6	None

**Table 2:** Marginal bone loss values in mesial and distal sites at different follow-up times (- loss/+gain) SD = standard deviation, Max. = maximum value, Min. = minimum value, n = number of implants.

Timepoint	Mean (mm)	SD (mm)	Median (mm)	Max. (mm)	Min. (mm)
All implants (n = 70)					
T1 – T0 (mesial)	0.115 mm	0.766 mm	0.015 mm	-2.98 mm	2.30 mm
T2 – T0 (mesial)	0.044 mm	0.744 mm	0.000 mm	-3.23 mm	2.14 mm
T3 – T0 (mesial)	-0.091 mm	0.787 mm	-0.070 mm	-2.93 mm	1.72 mm
T1 – T0 (distal)	0.166 mm	0.784 mm	0.100 mm	-1.97 mm	2.02 mm
T2 – T0 (distal)	0.130 mm	0.828 mm	0.025 mm	-2.51 mm	3.02 mm
T3 – T0 (distal)	-0.045 mm	0.819 mm	-0.090 mm	-3.11 mm	2.19 mm
Smoking patients (n = 40 implants)					
T1 – T0 (mesial)	0.218 mm	0.929 mm	0.060 mm	-2.98 mm	2.30 mm
T2 – T0 (mesial)	0.093 mm	0.893 mm	0.005 mm	-3.23 mm	2.14 mm
T3 – T0 (mesial)	0.015 mm	0.833 mm	0.010 mm	-2.93 mm	1.72 mm
T1 – T0 (distal)	0.216 mm	0.892 mm	0.100 mm	-1.97 mm	2.02 mm
T2 – T0 (distal)	0.122 mm	0.946 mm	0.015 mm	-2.51 mm	2.11 mm
T3 – T0 (distal)	0.042 mm	0.996 mm	-0.005 mm	-3.11 mm	2.19 mm
Non-smoking patients (n = 30 implants)					
T1 – T0 (mesial)	-0.024 mm	0.446 mm	-0.020 mm	-1.14 mm	1.46 mm
T2 – T0 (mesial)	-0.021 mm	0.488 mm	-0.070 mm	-0.88 mm	1.32 mm
T3 – T0 (mesial)	-0.232 mm	0.712 mm	-0.255 mm	-1.89 mm	1.39 mm
T1 – T0 (distal)	0.098 mm	0.619 mm	0.035 mm	-0.98 mm	1.90 mm
T2 – T0 (distal)	0.141 mm	0.656 mm	0.075 mm	-0.54 mm	3.02 mm
T3 – T0 (distal)	-0.162 mm	0.485 mm	-0.215 mm	-1.26 mm	0.93 mm

Using the Shapiro-Wilk test, the data distribution was non-parametric, as evidenced by P-values of less than 0.05. The marginal bone loss in the mesial and distal sites was statistically significantly different at the different time points using the Friedman test, P values = 0.00004 and 0.00001, respectively. Pairwise signed-rank test between time points revealed a statistically significant difference in marginal bone loss in all comparisons except T1 - T0 vs T2 - T0 on the distal side (P-value = 0.2). Using the two-way mixed ANOVA test, there was no statistically significant two-way interaction between smoking and time on marginal bone loss values in mesial and distal sites, P-values = 0.334 and 0.132, respectively.

## Discussion

This case series aimed to present seven patients with multiple non-restorable teeth or generalized periodontitis indicated for extraction and IFFR using the new CDSS introduced by Carames [8] to determine the number, position, distribution, and treatment options for implant placement according to the level of bone atrophy in the maxilla or mandible. The Carames classification includes five classes for each jaw according to the different levels of bone atrophy and bone level measurements. Most cases in our case series had a classification of CC1 or CC2, except for one case with a CC5 in the maxilla. Treatment options for fixed rehabilitations in each class are based on deciding factors, as Carames proposed. The deciding factors in choosing treatment options A or B include arch size, presence of natural teeth, parafunctional habits, maxillary sinus geometry in cases where closed sinus lifting or tilting of implants could be an option, and the remaining bone quality [8]. Using angled implants in CC2 classification treatment option B prevents distal cantilever in cases of limited bone height at first molar sites in the mandible or maxilla. According to the arch size and extension, the use of eight or six implants in the maxilla or mandible is proposed, as some cases had eight or six implants in the arch.

In the case with CC5 classification, the maxillary arch suffered from severe atrophy, with bone heights of less than 8 mm (anterior) and 4 mm (posterior) and widths of less than 6 mm. Treatment option B was chosen to allow immediate loading without a grafting procedure, with four short implants placed in the anterior region of the maxilla, two implants at the lateral incisors position, and two implants adjacent to the maxillary sinus wall. Two additional zygomatic implants were placed in the posterior region angled forward to obtain anchorage and stability from the zygomatic bone [8]. All implants placed had primary stability of 40 to 45 Ncm to allow successful immediate loading [13]. This CDSS by Carames offered a more patient-centered and straightforward way of choosing treatment plans for immediate IFFR in the maxilla or mandible.

The dual-zone bone grafting technique was used in all the cases after implant placement by grafting the jumping gap until the free gingival margin, which was shown to have promising results in peri-implant marginal tissue health and minimal loss of marginal bone [11,14]. After placement, the intraoral welding of implants allowed more stability and placement of a snugly fit provisional screw-retained denture that compressed the gingival tissues to prevent any gap formation and preserve the soft and hard tissues for healing. The vertical occlusal dimensions, smile line, and occlusion were adjusted while fabricating the provisional denture to allow for functional loading and minimize modifications needed in the final prosthesis. This was helpful because the provisional denture served as a guide to creating the definitive prosthesis by obtaining a biocopy [15]. Intraoral welding of implants has another advantage in immediate implant loading. It provides rigid fixation and reinforces the acrylic provisional denture, which is crucial for stress distribution. This helps keep the implants' micro-movements within the acceptable limit of 100 micrometers. These micro-movements can enhance the osteointegration process and prevent the risk of fibrous encapsulation of the implants if they fall within acceptable limits [16,17]. The study assessed outcomes based on the recently published ID-COSM [10], reflecting the benefits and harms of implant interventions and focusing on peri-implant marginal tissue health status as a significant outcome in IFFR.

In the current study, no implant loss or prothesis loss was reported during the three-year follow-up period, which agrees with the findings of a retrospective study by Carames et al. in 2021 [9] that analyzed implant survival in immediate implant-supported fixed complete dentures with a total of 882 patients receiving 6042 implants using the CDSS proposed by Carames [8]. They also considered the level of alveolar atrophy and different treatment options for the various classes. The authors found that the five-year cumulative implant survival rate was 97.9%. They observed that the survival rates for implants in the maxilla were significantly lower than those in the mandible. These survival rates decreased consistently with increasing alveolar atrophy, particularly in the maxilla. However, this finding was not as noticeable in the present study due to the smaller number of patients and implants with CCI or CCII classes.

Regarding the peri-implant marginal tissue health status, the findings indicated good peri-implant soft tissue health, no bleeding or suppuration on probing, and PPD in the normal range of 3 to 3.5 mm. As a change of baseline, the marginal bone loss values showed statistically significant differences in all time points (except T0-T2 vs. T0-T1 on the distal side), revealing minimal marginal bone loss during the three years in the distal and mesial sides ranging from +0.166 to -0.045 (mean values). Factoring in the smoking status as smoker or non, the two-way mixed ANOVA test revealed no significant interaction of smoking and time on marginal bone loss values. However, this finding is different from the previous studies [9,18-20] that indicated smoking as a risk factor for more marginal bone loss and more implant failure. However, this disagreement could be due to the smaller number of patients and implants placed and the lesser alveolar atrophy in classes CCI and CCII. Some cases showed more bone loss in smokers, with a maximum of -3.23 and -3.11 mm in mesial and distal sites, respectively. However, smoking did not significantly affect marginal bone loss values.

Some of the limitations in this current study include fewer patients and fewer implants placed compared to other studies. However, this study indicated promising peri-implant marginal tissue health with no implant loss in three years.

## Conclusions

Implant-supported fixed full-arch rehabilitation using the Carames classification as a clinical decision support system showed promising results in peri-implant marginal tissue health and no implant loss during three years of follow-up with a guide for different treatment options considering the bone level atrophy in both jaws. The implant placement and prosthesis fabrication protocol in this study could be of value in further research as it showed promising results.
